# Response rates and selection problems, with emphasis on mental health variables and DNA sampling, in large population-based, cross-sectional and longitudinal studies of adolescents in Norway

**DOI:** 10.1186/1471-2458-10-602

**Published:** 2010-10-12

**Authors:** Espen Bjertness, Åse Sagatun, Kristian Green, Lars Lien, Anne Johanne Søgaard, Randi Selmer

**Affiliations:** 1 Section for Preventive Medicine and Epidemiology, Institute of General Practice and Community Medicine, Faculty of Medicine, University of Oslo, Box 1130 Blindern, 0318 Oslo, Norway; 2 Tibet University Medical College, No. 1 South Luobulinka Road, Lhasa, 850002 Tibet, China; 3 Research Department, Centre for Child and Adolescent Mental Health, Eastern and Southern Norway, Box 4623 Nydalen, 0405 Oslo, Norway; 4 Institute of Psychiatry, University of Oslo, Box 1130 Blindern, 0318 Oslo, Norway; 5 Innlandet Hospital Trust HF, DPS Gjøvik, Kyrre Grepps 22, 2819 Gjøvik, Norway; 6 Division of Epidemiology, Norwegian Institute of Public Health, Box 4404 Nydalen, 0403 Oslo, Norway

## Abstract

**Background:**

Selection bias is a threat to the internal validity of epidemiological studies. In light of a growing number of studies which aim to provide DNA, as well as a considerable number of invitees who declined to participate, we discuss response rates, predictors of lost to follow-up and failure to provide DNA, and the presence of possible selection bias, based on five samples of adolescents.

**Methods:**

We included nearly 7,000 adolescents from two longitudinal studies of 18/19 year olds with two corresponding cross-sectional baseline studies at age 15/16 (10^th ^graders), and one cross-sectional study of 13^th ^graders (18/19 years old). DNA was sampled from the cheek mucosa of 18/19 year olds. Predictors of lost to follow-up and failure to provide DNA were studied by Poisson regression. Selection bias in the follow-up at age 18/19 was estimated through investigation of prevalence ratios (PRs) between selected exposures (physical activity, smoking) and outcome variables (general health, mental distress, externalizing problems) measured at baseline.

**Results:**

Out of 5,750 who participated at age 15/16, we lost 42% at follow-up at age 18/19. The percentage of participants who gave their consent to DNA provision was as high as the percentage that consented to a linkage of data with other health registers and surveys, approximately 90%. Significant predictors of lost to follow-up and failure to provide DNA samples in the present genetic epidemiological study were: male gender; non-western ethnicity; postal survey compared with school-based; low educational plans; low education and income of father; low perceived family economy; unmarried parents; poor self-reported health; externalized symptoms and smoking, with some differences in subgroups of ethnicity and gender. The association measures (PRs) were quite similar among participants and all invitees, with some minor discrepancies in subgroups of non-western boys and girls.

**Conclusions:**

Lost to follow-up had marginal impact on the estimated prevalence ratios. It is not likely that the invitation to provide DNA influenced the response rates of 18/19 year olds. Non-western ethnicity, male gender and characteristics related to a low social class and general and mental health problems measured at baseline are associated with lost to follow-up and failure to provide DNA.

## Background

The general decline in the participation rate in epidemiological studies during recent years [1] may introduce errors in the estimations of exposure and disease occurrence and association measures, and is a major concern for researchers. There are several epidemiological studies investigating factors associated with non-response among adolescents, but studies on the effect of self-selection on association measures, both in cross-sectional and longitudinal designs, are scarce. It is difficult to find discussions of response rates in *genetic *epidemiological studies and the factors associated with non-response or refusal of agreeing to DNA sampling, even though the same threats to internal validity are in operation as for studies with non-genetic information. This is a concern, as the proportion of epidemiological studies that collect biological specimens increases over time, as reviewed by Morton et al. [2]. This review further reported that less than one-third of epidemiological studies yielded separate participation figures for the biological specimen component of the study. Additionally, we have been able to disclose only one study which has investigated a possible selection bias in the association between genotype and outcome [3], and no studies on factors associated with a refusal to agree to DNA sampling.

Studies of pre-adolescents [4] and adults [5,6] concluded that selection has little or no impact on the association measures, although such information is insufficient, especially in adolescents and children.

As compared with postal-based, school-based research provides a comparatively inexpensive method of obtaining large samples of children and adolescents with high response rates. In surveys of adolescents under the age of 16, parental consent is required in several countries, including Norway. Tiggers [7] reviewed that active parental consent led to parental permission and response rates in the range of 30%-60% for students biased in the direction of an exclusion of minorities, students having problems in school or students engaged in or at risk for problem behaviours. If passive parental consent is required, parental permission is in the range of 93%-100% [7].

Non-genetic epidemiological studies have reported that adolescents with characteristics associated with poor health have the lowest likelihood of participating in health surveys. Those included lower social status [8]; low maternal education or income [9,10 (young adults)]; being born in a third-world country [11] and those with a less favourable lifestyle [12], including substance abuse [13] and smoking [10 (young adults), [14-16]]. Furthermore, adolescents with a young mother [10 (young adults)]; with less favourable [17-19] or more favourable [20] relations with peers; living in urban areas [10,21]; with diagnosed ADHD [22]; low cognitive performance [4,19,23]; higher levels of psychopathology [19,24,25] and serious psychiatric illness [26] had the lowest participating rate in health surveys.

The aims of the present genetic epidemiological study of adolescents aged 15/16 and 18/19 in the multi-cultural city of Oslo and the rural county of Hedmark were to:

1. Describe response rates across genetic and non-genetic epidemiological studies;

2. Identify predictors of lost to three-year follow-up in a genetic epidemiological study of 18/19-year-old adolescents, with a particular emphasis on gender and ethnicity;

3. Identify predictors of failure to provide DNA in a follow-up of 18/19-year-old adolescents;

4. Investigate the magnitude and direction of possible selection bias in a follow-up at age 18/19 through an investigation of association measures (prevalence ratio) between selected exposures and general and mental health outcome variables measured at baseline (aged 15/16).

## Methods

### 15/16 year olds in 2001: two cross-sectional studies of 10^th ^graders in Oslo and Hedmark

**Figure 1:**
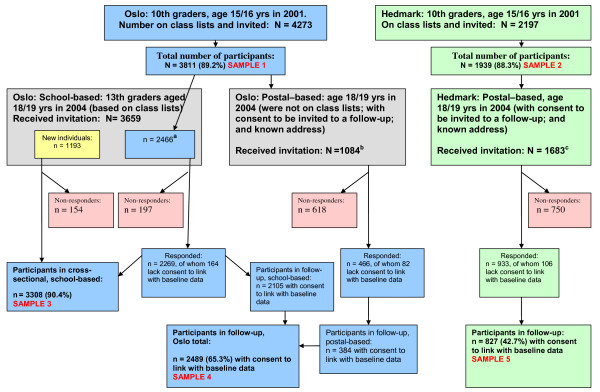
**Flow chart of the study populations**. ^a^27 individuals had an unknown address for the postal-based reminder; ^b^61 individuals had an unknown address and 173 did not consent to be invited to a follow-up; ^c^27 had an unknown address and 229 did not consent to be invited to a follow-up. An additional 55 who gave their consent, but did not fill out the questionnaire at baseline, were invited, of whom 18 participated. They are defined as non-responders at baseline, and are not included in the present analyses.

All students in 10th grade in all 60 and 41 primary schools in Oslo (Figure 1, Sample 1) and Hedmark (Figure 1, Sample 2), respectively, were invited to enter the youth portion of the Oslo Health Study and the Hedmark Health Study, respectively. The data collection was performed at the end of the school year in 2001. All parents received written information and the students signed a consent form before beginning participation. The students completed two four-page questionnaires during two school hours. A project assistant was present in the classroom to inform the students about the survey and to administer the questionnaires. Questionnaires were left at school to be completed by students not present on the day of the survey. Those who did not respond received a copy by mail to their home address, together with a pre-stamped return envelope. Ten years at school is compulsory in Norway; hence, the study included all 15/16 year olds in the study areas. A more detailed description has been published elsewhere [27].

### 18/19 year olds in 2004: one cross-sectional study of 13^th ^graders in Oslo and two longitudinal studies in Oslo and Hedmark

A three-year follow-up study of the two cross-sectional samples of 10th graders in Oslo (Figure 1, Sample 4) and Hedmark (Figure 1, Sample 5) was conducted in 2004, partly in school and partly via mail. In both instances, the participants were invited to give consent to and provide DNA through a sampling of cells from cheek mucosa. The data collection was performed before the end of the school year, and the participants were invited to join a lottery with three sums of NOK 15,000 (i.e. USD 2,470/EUR 1,740).

In Oslo, the follow-up was conducted as a school-based study similar to the baseline, inviting all the final year students (13^th ^graders) in all 32 secondary high schools. This was done in order to reach as many of the baseline participants as possible, representing a cross-sectional study of 13^th ^graders in Oslo (Figure 1, Sample 3). In Norway, all young people between the ages of 16 and 19 have a right to three years of upper secondary education and training funded by the government, and the vast majority of them take advantage of this opportunity. The students filled in a four-page questionnaire and provided a cell sample from their cheek mucosa during one school hour. Because a number of students were not present when the study was conducted, schools were visited several times. Those students who were not reached in school were invited by mail and included in the school-based portion of the follow-up study.

Participants from the baseline study who were not enrolled in the final year of secondary high school in Oslo and who had consented to participate in a follow-up were invited by mail (Figure 1). The package included an invitation letter, an information brochure, a consent form, a questionnaire and two cytobrushes, including a container for buccal cell sampling and a pre-stamped return envelope. Two reminders were sent to those who did not respond.

Methods similar to those applied in the *postal-based portion *in Oslo were applied for the entire three-year follow-up of all participants from the 2001 baseline study in rural Hedmark (Figure 1).

The youth studies are described more thoroughly elsewhere [28].

## Measures

### DNA

Two samples of cells from the cheek mucosa were collected from both the left and right side, using two cytobrushes (Medscand Medical AB, Malmö, Sweden) in the studies of 18/19 year olds. In the school-based study, the students were instructed on how to perform the rubbing, which was done simultaneously by all students in the classroom. In the postal-based studies an instruction letter was included, and a plastic tube with a cap and two brushes was returned by mail together with the questionnaire and consent form. The cytobrushes were frozen at -20 degrees C.

### Mental health

#### Externalizing problems

We used the self-report version of the Strengths and Difficulties Questionnaire (SDQ) [29,30], which is a 25-item wide-angle screening questionnaire with five subscales. Each subscale consists of five items, generating scores for emotional symptoms, conduct problems, hyperactivity-inattention, peer problems and prosocial behaviour. Each item can be answered with "not correct" (0), "partly correct" (1) or "completely correct" (2). We used two of the SDQ subscales: conduct problems and hyperactivityinattention to summarize an index of externalizing problems with a cut-off point at the 90th percentile of the study sample [31].

The SDQ self-report is designed and validated for youngsters (11-16 years old), but has also been used as a valuable instrument for older youths [32,33]. To adapt the instrument to 18/19 year olds, some small linguistic changes were made in the follow-up questionnaire in accordance with the approved Norwegian translation.

#### Mental distress

The Hopkins Symptom Checklist (HSCL-10) is comprised of 10 questions regarding psychological symptoms of depression and anxiety (mental distress) experienced the previous week [34]. For each question, there are four possible answers ranging from: "not troubled" (1) to "heavily troubled" (4), and the average score of the items is used as a measure of mental distress [34]. The 10-item version has approximately the same sensitivity and specificity for detecting psychological symptoms or global distress as the more widely used HSCL-25 [35-37]. The HSCL-25 version has proven to have a satisfactory validity and reliability as a measure of mental distress in adults [38], and the 10-question version performs almost as well as the longer versions, including among subjects aged 16-24 [34]. An average score for all 10 items equal or above 1.85 was used as the cut-off point for mental distress, corresponding to the 1.75 cut-off of HSCL-25 [34].

### General health

Self-evaluated general health status was measured from the question: How would you describe your present state of health? (poor, not very good, good, very good). The categories were operationalized into *poor/not very good *and *good/very good*.

### Physical activity in leisure time

Physical activity was measured by a question on the amount of weekly hours concerning physical activity outside of school "to an extent that made you sweat and/or out of breath". The answers were rated 0, 1-2, 3-4, 5-7, 8-10 or 11 hours or more per week, and were operationalized in the present study into *0-2 hours per week *and *more than 2 hours*.

### Smoking

The question: "Do you smoke or have you smoked earlier?" had four alternatives: never smoking; smoking before, but quit; smoking now and then; and smoking daily. The two middle categories were merged together into one category since the initial results showed that these two groups responded in a similar way.

### Ethnic background/Country of origin

Ethnicity was self-reported and determined on basis of the parents' country of birth. Statistics Norway's definition of ethnic minorities, which is those having both parents born in a country other than Norway, was applied [39]: western (one or both parents born in Norway or another western country) vs. non-western (both parents born in a non-western country).

### Parental educational level, income and marital status

To obtain information on the parental educational level and income, the questionnaire data was linked to socio-demographic information collected by Statistics Norway for all participants [40,41]. We applied Statistics Norway's register of highest parental education completed as per Oct. 1, 2000.

The educational level was operationalized into three major groups according to the highest attained educational level: *university/college*; *higher secondary *and *lower secondary *education for the father. Income was operationalized into high (above the 75^th ^percentile), medium (25^th ^to 75^th ^percentile) and low (below the 25^th ^percentile) for the father. The family economic status was self-reported as *bad*, *medium*, *good *or *very good*, based on a question comparing the family economy with other families in Norway.

The marital status of the parents was dichotomized into *married *(married/living together) versus *not married *(unmarried/divorced/separated/one or both parents deceased).

### Educational plans

The 15/16 year olds recorded the highest future education they had considered, which was operationalized into: *university higher *(i.e. university or regional college higher degree); *other *(university or regional college intermediate level; upper secondary school; vocational education at upper secondary school; one year at upper secondary school; other plans) and *not decided*. Their educational ambition was used as an indicator of social class of destination.

### Invitation group

We created a variable "invitation group", dividing participants into three groups based on participation using "*postal-based Hedmark*", *postal-based Oslo*" and "*school-based Oslo*" as an exposure variable and for adjustment in multivariate analyses.

### Statistical methods

Response rates are presented as numbers and percentages for the five studies (Figure 1, Samples 1-5). For all studies of 18/19 year olds in 2004, the numbers and percentages for agreeing to link data to other health surveys and registers, as well as agreeing to provide DNA, are presented. Additionally, for the two longitudinal studies, response and consent rates are presented by gender and ethnicity. The relative risk (RR_crude _and RR_adjusted_) with a corresponding 95% Confidence Interval (CI) for lost to follow-up in 2004 is presented for baseline socio-demographic characteristics (in 2001), using Poisson regression analyses. Data from the two study sites (Hedmark and Oslo) are combined, as there were only small differences in results between the two samples, and we have adjusted for "invitation groups". The variables predicting "lost to follow-up" are similar to those predicting "failure to provide DNA". Thus, we do not present results for "failure to provide DNA", but regard analyses of "lost to follow-up" as a proxy.

Crude and adjusted relative risks (RRs), with a 95% CI of lost to follow-up for selected baseline variables, general health, mental health and risk factors, are presented. Adjustments were done for invitation group, gender, ethnicity, family economy, parental marital status, educational plans and father's education and income using Poisson regression.

The possible effects of selection in attendance on the *associations *were also assessed based on baseline data. The association (prevalence ratio, PR) between selected risk factors (smoking, physical activity) and three outcome variables (general health, mental distress and externalizing symptoms) are presented by gender and ethnicity among both the participants and all invitees. Analyses were conducted separately by gender and ethnicity due to the large differences in the occurrence of mental health problems. The independent variables were chosen since they are well-known risk factors for adverse health effects and one of them (smoking) is associated with non-response, while the other (physical inactivity) is not.

Analyses were performed using SPSS version 14 and STATA version 10.

### Ethics

The study protocol was evaluated by the Regional Committee for Medical Research Ethics and approved by the Norwegian Data Inspectorate. The studies carried out in the schools received approval from the school authorities.

Information from public registers in Statistics Norway about the father's education and income was linked with data from the questionnaire/studies through the individual's personal identification number. All personal identification was erased before the data were analysed.

## Results

The samples constituting the present study include data from two cross-sectional school-based studies with a total of 5,750 (88.9% response) 15/16-year-old 10^th ^graders in Oslo (Sample 1, Figure 1) and Hedmark (Sample 2, Figure 1) obtained in 2001, and one corresponding cross-sectional study of 3,308 (90.4% response) 18/19-year-old 13^th ^graders in Oslo (Sample 3, Figure 1) obtained in 2004 (DNA from 3,095) (Table 1). Additionally, we have data on 18/19 year olds from the postal follow-up studies in Oslo (n = 466) and Hedmark (n = 933) in 2004, yielding questionnaire data on a total of 4,707 18/19 year olds, with DNA from 4,305 individuals (Table 1). Finally, based on two longitudinal studies from Oslo and Hedmark, we have three-year follow-up data of a total of 3,668 (70.1% response) 18/19 year olds, with DNA from 3,355 individuals, of whom 3,316 agreed to linkage to the baseline data (Sample 4 and 5, Figure 1). All data obtained in the latter two longitudinal studies is derived from some of the participants in the cross-sectional studies mentioned above (Table 1).

**Table 1:** Number of invitees and number of participants in the cross-sectional studies in 2001 and 2004, and three-year follow-up of samples from 2001

	Invitees	Participated		n and % of participants accepting linkage of data to other health surveys and registers		n and % of participants accepting DNA (Cyto-brush)	
Study	N	n	%	n	%	n	%
							
**Cross-sectional data collections**							
							
*15/16 years old, 2001*							
Oslo, school based **(sample 1)**^1^	4273	**3811**	89.2	3433	90.1		
Hedmark, school-based **(sample 2)**^1^	2197	**1939**	88.3	1791	92.4		
TOTAL, Oslo and Hedmark	6470	5750	88.9	5224	90.9		
							
*18/19 year old, 2004*							
Oslo, school-based **(sample 3)**^1^	3659	**3308**	90.4	3036	91.8	3095	93.6
							
**Three years follow-up of samples from 2001**							
							
*18/19 years old, 2004*							
Oslo total **(sample 4)**^1^	3550	2735	77.0	**2489**	91.0	2527	92.4
School-based	2466^2^	2269	92.0	2105	92.8	2145	94.5
Postal-based	1084^3^	466	43.0	384	82.4	382	82.0
Hedmark, postal-based **(sample 5)**^1^	1683^4^	933	55.4	**827**	88.6	828	88.7
TOTAL, Oslo and Hedmark	5233	3668	70.1	3316	90.4	3355	91.5

Number of participants who agreed to link their data to other health studies and registries and number who agreed to DNA analyses.

^1^Refers to samples 1-5 in the float chart (Figure 1);

^2^1193 individuals did not take part in the baseline study in 2001 (935 were new individuals and 258 individuals took part in a similar study in 2000). This explains why there are 1193 less individuals invited to the follow-up of the Oslo, school-based, as compared with the cross-sectional, school-based;

^3^173 individuals out of 3811, who participated in the baseline survey in Oslo in 2001 and were not on school lists in 2004, did not consent to a new invitation, in addition 61 had unknown address, thus 1084 were invited by post to the follow-up in 2004;

^4^229 individuals out of 1939 who participated in the baseline survey in Hedmark in 2001 did not consent to a new invitation, in addition 27 had unknown address, thus 1683 were invited in 2004.

### Response rates

In the studies of 10^th ^graders aged 15/16, in which passive parental consent was obtained, the participation rate was similar to rural Hedmark (88.3%) and urban Oslo (89.2%) (Table 1). The response rate was also quite similar in the cross-sectional school-based study of 13^th ^graders aged 18/19 (90.4%), although collection of DNA from each individual was added as part of the survey. The response rate in the pure postal-based study in Hedmark among 18/19 year olds was considerably lower (55.4%) (Table 1). A total of 2,489 (70.1%) out of 3,550 invited 18/19 year olds in Oslo participated in the follow-up and gave their consent to linkage, while in Hedmark 827 (49.1%) out of 1,683 of those invited did so.

Almost all *participants *in the school-based survey of 18/19 year olds consented to give their DNA (93.6%), and the rate was also high in the postal-based data collections, at 82% and 88.7% in Oslo and Hedmark, respectively (Table 1). The percentage of participants who consented to give their DNA was about the same as the percentage who agreed to link their data to registers and previous health surveys.

### Factors associated with follow-up rates

Lost to follow-up was closely related to *failure to provide DNA*, and analyses revealed that there were similar predictors operating in the two instances. As a result, the factors associated with lost to follow-up presented in the following are valid for *failure to provide DNA *(data not shown).

For the purpose of linking data from the two time points, 2,489 (65.3%) out of 3,811 15/16 year olds in Oslo who participated in the baseline study in 2001participated and consented to the linkage of data in 2004 (Table 2). Thus, the *lost to follow-up *in Oslo was 34.7%. In Hedmark, 827 (42.7%) of the participants in 2001 also participated and agreed to linkage in 2004, yielding a *lost to follow-up *of 57.3%. More girls than boys and more participants with a Norwegian/western than non-western background participated in the follow-up study (Table 2). In Oslo, 97.7% of participants in the follow-up who consented to linkage of data also agreed to provide DNA, which was quite similar to Hedmark (96.3%) (Table 2).

**Table 2:** Number of participants in the longitudinal studies^1 ^in Oslo and Hedmark according to gender and ethnicity

		Baseline 2001	Follow-up 2004			
		
		Participants	Participated and accepted linkage of **data**^**2**^		Participants who accepted linkage of **data**^**2 **^**and provide DNA**^**3**^	
		N	n	%	n	%
Oslo						
Total^4^		3811	2489	65.3	2433	97.7
Ethnicity^6^:						
Norway/western	Girls	1424	1103	77.5	1078	97.7
	Boys	1456	906	62.2	893	98.6
Non-western	Girls	422	261	61.8	249	95.4
	Boys	425	192	45.2	186	96.9
						
Hedmark						
Total^5^		1939	827	42.7	796	96.3
Ethnicity^7^:						
Norway/western	Girls	950	494	52.0	477	96.6
	Boys	931	317	34.0	303	95.6
Non-western	Girls	17	6	35.3	6	100.0
	Boys	25	9	36.0	9	100.0

^1^Participated both in the baseline study 2001 and in the follow-up 2004;

^2^Participants accepting linkage of data to other health surveys and registers, i.e. linkage of data to the baseline data;

^3 ^Percent providing DNA among participants accepting linkage of data; ^4,5^Due to missing data in Oslo on gender and/or ethnicity (84), and in Hedmark on ethnicity (16), the numbers will not sum up to the total numbers in Oslo, nor Hedmark; ^6,7^Based on self-reported native country of mother and father.

There were no major differences between Oslo and Hedmark in *socioeconomic *predictors for lost to follow-up (data not shown). Therefore, the relative risk of selected socio-demographic baseline factors for lost to follow-up for Hedmark and Oslo are presented as combined (Table 3). Significant predictors were: male gender; non-western ethnicity; postal survey compared to school-based; lower educational plans than university/higher education; low education and income of father; low perceived economy in the family and unmarried as compared to married parents (Table 3). Adjustments for "invitation group" resulted in weaker risk estimates, except for non-western ethnicity which increased from RR = 1.17 (95% CI: 1.08-1.26) to RR = 1.31 (95% CI: 1.21-1.43). Separate analyses by western/non-western ethnicity were conducted. There was no major change in risk estimates for western boys and western girls (results not shown). However, in non-western boys and girls, the father's income or education was not a significant predictor for lost to follow-up, and a poor perceived family economic situation was significantly associated with lost to follow-up in girls only (results not shown).

**Table 3:** Baseline socio-demographic characteristics in 2001, prevalence and relative risk (RR) of lost to follow-up in 2004 in Oslo and Hedmark combined

Variables collected	Base-line	Lost to follow-up		Relative risk of lost to follow-up					
				
In 2001	N	n	%	**RR**_**crude**_^**1**^	95% CI		**RR**_**adj**_^**1,2**^	95% CI	
Gender^3^									
Boys	2894	1455	50	Ref			Ref		
Girls	2835	958	34	0.67	0.63	0.72	0.72	0.67	0.77
									
Ethnicity^4^									
Western	4768	1948	41	Ref			Ref		
Non western	896	428	48	1.17	1.08	1.26	1.31	1.21	1.43
									
Invitation group									
Oslo school	2493	388	16	Ref					
Oslo post	1145	761	66	4.27	3.86	4.72			
Hedmark post	1710	883	52	3.32	3.00	3.68			
									
Education plans									
University/higher	2847	911	32	Ref			Ref		
Other	1940	1119	58	1.80	1.69	1.93	1.23	1.15	1.33
Not decided yet	859	336	39	1.22	1.11	1.35	1.07	0.96	1.18
									
Family economic situation									
Bad	184	108	59	Ref			Ref		
Average	1824	847	46	0.79	0.69	0.90	0.86	0.75	0.99
Good	3014	1155	38	0.65	0.57	0.74	0.82	0.71	0.94
Very good	620	266	43	0.73	0.63	0.85	0.95	0.81	1.12
									
Education father									
Lower secondary	786	381	48	Ref			Ref		
Upper secondary	2470	1082	44	0.90	0.83	0.98	0.97	0.89	1.05
University/College	1837	471	26	0.53	0.48	0.59	0.79	0.71	0.87
									
Income father									
Low	1190	585	49	Ref			Ref		
Medium	2654	1011	38	0.77	0.72	0.84	0.84	0.78	0.90
High	1161	304	26	0.53	0.48	0.60	0.80	0.72	0.88
									
Parents' marital status									
Married	3891	1518	39	Ref			Ref		
Not married	1806	876	49	1.24	1.17	1.32	1.13	1.06	1.21

^1^When substituting "lost to follow-up" with "failure to provide DNA" as dependent variable, the RRs were similar;

^2^Adjusted for invitation group;

^3^Gender does not sum up to 5750 as shown in Figure 1 and Table 1 due to "missing information on gender" on 21 individuals;

^4^Based on self-reported native country of mother and father.

The relative risk of selected *baseline health and health-related factors *for lost to follow-up did not differ in separate analyses between Hedmark and Oslo. In the combined data set, poor self-reported health (borderline significantly), externalized symptoms and smoking were significant predictors for lost to follow-up (Table 4). When stratifying ethnic groups, none of the selected factors were significant predictors for lost to follow-up in non-western boys and girls (data not shown).

**Table 4:** Baseline health characteristics in 2001, prevalence and relative risk of lost to follow-up in 2004 in Oslo and Hedmark combined

Variables collected	Base-line	Lost to follow-up		Relative risk of lost to follow-up					
				
In 2001	N	n	%	**RR**_**crude**_^**1**^	95% CI		**RR**_**adj**_^**1,2**^	95% CI	
Self-reported health									
Good/very good	4951	2057	42	Ref			Ref		
Bad/not so good	706	341	48	1.16	1.07	1.26	1.09	0.98	1.20
Total	5657	2398	42						
									
Mental distress/HSCL above cutt-off									
No	4695	1961	42	Ref			Ref		
Yes	971	415	43	1.02	0.94	1.11	1.06	0.96	1.16
Total	5666	2376	42						
									
Externalized symptoms									
No	4985	1990	40	Ref			Ref		
Yes	702	399	57	1.42	1.32	1.53	1.18	1.08	1.28
Total	5687	2389	42						
									
Physical activity									
3+ h	3556	1426	40	Ref			Ref		
0-2 h	2041	913	45	1.12	1.05	1.19	1.03	0.95	1.10
Total	5597	2339	42						
									
Smoking									
Never or stopped	3962	1538	39	Ref			Ref		
Occasionally or daily	1749	871	50	1.28	1.21	1.36	1.19	1.11	1.28
Total	5711	2409	42						

^1^When substituting "lost to follow-up" with "failure to provide DNA" as dependent variable, the RRs were similar

^2^Adjusted for invitation group, gender, ethnicity, family economy, parental marital status, educational plans, father's education and income. The number included in adjusted analyses is lower than in crude analyses due to missing data for some of the variables. For example, in analyses of self-reported health there are 5657 in crude analyses and 4527 in adjusted analyses. In particular, we lack data on father's education and income, which are obtained from Statistics Norway. In reanalyses of RR_crude_, including only the number available in adjusted analyses, the associations were slightly stronger (except for physical activity), but did not change confidence intervals to significantly/non-significantly for either of the associations.

In subgroups of western boys, the predictors were similar to the total sample, with mental distress yielding a significant effect, while in western girls only externalized symptoms and smoking were significant predictors for lost to follow-up (data not shown).

### Association measures

In order to investigate a possible distortion in association measures due to lost to follow-up, we examined the prevalence ratios (PRs) between baseline exposure data from 2001 (smoking and physical activity) and selected outcome health variables (self-reported health, mental distress and externalized symptoms) among participants and all invitees in the follow-up in 2004.

Regarding the associations between *physical activity *and self-reported health, mental distress and externalized symptoms, the prevalence ratios were similar in the groups of participants and all invitees except for self-reported health among non-western girls, in which the PR was 3.0 (1.3-6.7) for participants and 2.0 (1.1-3.6) for all invitees (Table 5). Even so, the number of participants in these groups was low and the confidence intervals were wide. The analyses of association between *smoking *and self-reported health, mental distress and externalized symptoms gave similar prevalence ratios in the groups of participants and all invitees for both western boys and girls (Table 6). In subgroups of non-western boys and girls, there were differences in PRs and CIs between participants and all invitees. Nevertheless, the number of participants in these groups was low and the confidence intervals were wide (Table 6).

**Table 5:** Associations between baseline physical activity and baseline self-reported health, mental distress and externalized symptoms among participants in 2004 and all invitees

	Participants 2004							All invitees						
		
**Gender/ethnicity**^**1**^	Physical activity 2001							Physical activity 2001						
Health variables collected	0-2 h		3+ h		Prevalence ratio			0-2 h		3+ h		Prevalence ratio		
in 2001	n	%	n	%	PR	95% CI		n	%	n	%	PR	95% CI	
														
Norway/western														
Boys														
Bad/not so good self-reported health	42	14.1	45	5.0	2.8	1.9	4.2	103	16.2	116	6.9	2.4	1.8	3.0
Mental distress - HSCL above cut off	27	8.9	51	5.6	1.6	1.0	2.5	78	12.2	127	7.5	1.6	1.2	2.1
Externalized symptoms	31	10.3	85	9.4	1.1	0.7	1.6	91	14.2	225	13.2	1.1	0.9	1.3
														
Girls														
Bad/not so good self-reported health	114	19.9	83	8.6	2.3	1.8	3.0	182	20.4	142	10.3	2.0	1.6	2.4
Mental distress - HSCL above cut off	140	24.4	215	21.8	1.1	0.9	1.3	235	26.3	318	22.6	1.2	1.0	1.3
Externalized symptoms	55	9.5	72	7.3	1.3	0.9	1.8	119	13.2	131	9.3	1.4	1.1	1.8
														
														
Non western														
Boys														
Bad/not so good self-reported health	6	10.9	11	7.7	1.4	0.5	3.7	21	13.6	24	8.6	1.6	0.9	2.7
Mental distress - HSCL above cut off	12	21.4	12	8.6	2.5	1.2	5.2	24	15.7	25	9.1	1.7	1.0	2.9
Externalized symptoms	9	16.4	15	10.6	1.5	0.7	3.3	24	16.0	36	13.3	1.2	0.7	1.9
														
														
Girls														
Bad/not so good self-reported health	38	22.2	6	7.5	3.0	1.3	6.7	61	21.0	12	10.3	2.0	1.1	3.6
Mental distress - HSCL above cut off	56	32.2	29	36.3	0.9	0.6	1.3	89	30.8	36	31.3	1.0	0.7	1.4
Externalized symptoms	19	10.9	8	9.9	1.1	0.5	2.4	33	11.2	10	8.7	1.3	0.7	2.5

Percentage with health symptoms and prevalence ratios (PRs) within categories of physical activity.

^1 ^Based on self-reported native country of mother and father.

**Table 6:** Association between baseline smoking habits and baseline self-reported health, mental distress and externalized symptoms among participants in 2004 and all invitees

	Participants 2004							All invitees						
		
**Gender/ethnicity**^**1**^	Smoking 2001							Smoking 2001						
Health variables collected	yes		no		Prevalence ratio			yes		no		Prevalence ratio		
in 2001	n	%	n	%	PR	95% CI		n	%	n	%	PR	95% CI	
														
Norway/western														
Boys														
Bad/not so good self-reported health	37	13.2	51	5.5	2.4	1.6	3.5	111	16.4	113	6.7	2.4	1.9	3.1
Mental distress - HSCL above cut off	30	10.6	48	5.2	2.1	1.3	3.2	100	14.8	107	6.3	2.3	1.8	3.0
Externalized symptoms	52	18.4	65	7.0	2.6	1.9	3.7	169	24.8	154	9.1	2.7	2.2	3.3
														
Girls														
Bad/not so good self-reported health	100	19.9	108	10.2	2.0	1.5	2.5	178	21.0	163	11.0	1.9	1.6	2.3
Mental distress - HSCL above cut off	167	33.1	193	17.9	1.9	1.6	2.2	287	33.6	278	18.6	1.8	1.6	2.1
Externalized symptoms	84	16.6	44	4.1	4.1	2.9	5.8	180	21.1	76	5.1	4.2	3.2	5.4
														
Non western														
Boys														
Bad/not so good self-reported health	5	14.7	12	7.4	2.0	0.8	5.3	20	19.8	28	8.2	2.4	1.4	4.1
Mental distress - HSCL above cut off	8	24.2	16	9.9	2.5	1.1	5.3	15	15.3	34	10.1	1.5	0.9	2.7
Externalized symptoms	6	17.6	18	11.1	1.6	0.7	3.7	26	26.3	36	10.8	2.4	1.5	3.8
														
Girls														
Bad/not so good self-reported health	11	26.2	35	16.0	1.6	0.9	3.0	20	28.2	56	15.6	1.8	1.2	2.8
Mental distress - HSCL above cut off	17	39.5	70	32.0	1.2	0.8	1.9	33	45.8	96	27.3	1.7	1.2	2.3
Externalized symptoms	13	30.2	14	6.3	4.8	2.4	9.4	23	31.9	21	5.9	5.4	3.2	9.3

Percentage with health symptoms and prevalence ratios (PRs) among smokers and non-smokers.

^1 ^Based on self-reported native country of mother and father.

## Discussion

### Main findings

We report similarly high response rates in all the school-based surveys - irrespective of age of the adolescents, year of the study or whether the survey was carried out in urban Oslo or rural Hedmark. In the follow-up, the response rate was markedly higher in Oslo when school-based and postal-based data collection were combined in comparison with the pure postal-based data collection in Hedmark. The rate of participants who gave their consent to DNA provision was as high as the rate of those who consented to linkage of data with other health registers and previous surveys. Significant predictors of lost to follow-up and failure to provide DNA samples were: male gender; non-western ethnicity; postal survey compared with school-based; lower educational plans than university/higher education; low education and income of father; low perceived economy in the family; unmarried as compared with married parents; poor self-reported health; externalized symptoms and smoking, with some differences in subgroups of ethnicity and gender. Regarding the association between selected exposures and outcomes, the main finding was that the association measures (PRs) were quite similar in the groups of participants and all invitees. In subgroups of non-western boys and girls, however, we found some differences, though the pattern was inconsistent, the number in the analysis was small and the confidence intervals of the estimated associations were large.

### Response rates

When conducting epidemiological studies, we aim to select samples in which all groups are represented in the study sample in the same way as their representation in the general population. Still, almost every study is hampered by a number of invitees who decline to participate. Any sign of selective attendance in which certain exposed groups are grossly under or overrepresented may incur disturbances to the conclusion. Epidemiological studies with a low level of participation are particularly vulnerable to self-selection bias threatening the internal validity. In the three present cross-sectional, school-based studies, the response rate was approximately 90%. In addition to the advantage with the classroom setting, several other factors may have contributed to the high response rates. Active parental consent was not needed, which in other studies has reduced the response rate to the level of 19%-60% [7,42]. We used no invasive methods and the data collection only took a little of the participants' time [43,44]: two hours for the 15/16 year olds and one hour for the 18/19 year olds. Face-to-face recruitment instead of a less personal form of contact between the study recruiter and potential participants may also increase the participation rate [45]. Among adults, monetary incentives may increase the response rate, but the effect on differential study participation is mixed [46]. Greater monetary incentives may have a greater impact on minority and low education individuals participating than on those who are non-minority with a higher education, though in contrast, potential responders with a high income or education may have a greater demand to be compensated for their time [46]. In the present study of 18/19 year olds, an incentive was given by letting participants join a lottery consisting of three sums of NOK 15,000 (i.e. USD 2,470/EUR 1,740), but it is not known whether incentives may bias studies of adolescents or had any impact on the response rate in this study.

### Predictors of lost to follow-up

About 10% did not agree to the linkage to other health surveys or registries, including their own baseline, thereby contributing to "lost to follow-up". In the consent form, the question of agreeing to a linkage to their own baseline was written in the same sentence as the linkage to registers. We are not able to rule out whether this mix could be the reason why so many adolescents refused the linkage.

Most of the predictors of lost to follow-up found in the present genetic epidemiological study have previously been reported in surveys of adolescents, and are the ones most often supportive of our findings [10,21]. In addition, we have found the following predictors of lost to follow-up, which as far as the authors are aware, have not previously been reported: postal survey compared with school-based; lower educational plans than university/higher education and low perceived economy in the family. Most studies report that urban area of living, as compared to rural, predict non-response [10,21], which is in contrast to the findings of the present study, in which we detected no differences between Oslo and Hedmark in the response rate among 15/16-year-old 10^th ^graders. It could be that school-based studies are less sensitive to location than other settings due to oral information about the purpose of the study [45] and a possible team feeling.

### Association measures

In the follow-up studies, the response rate was 65% in Oslo when combining the school-based and postal portion and only 43% in Hedmark, which is a concern regarding internal validity. However, in a respiratory health survey in Norway of 15-70-year olds, early responders were compared with late responders after a first and second reminder and telephone follow-up with respect to prevalence estimates and association measures [6]. The response rates increased from 42.7% to 79.9%, but there were only marginal differences in the exposure-disease relationship and prevalence estimates when initial responders were compared with all responders. This is in accordance with the present study of adolescents, in which we found no marked differences in association measures (PRs) among responders and all invitees, when restricted to western girls and boys. In non-western girls and boys, however, there were differences in the prevalence ratios in the association between exposure to smoking and physical activity on selected outcomes, with no discernable pattern. This could be due in part to the low number of participants in these groups or information bias, i.e. a linguistic or cultural problem in understanding the meaning of any of the questions from the questionnaire [47,48].

We may also draw support from a Dutch study on pre-adolescents by de Winter et al. [4] regarding western boys and girls in the present study, as well as a warning that prevalence estimates of mental health problems may increase with increasing participation rates. They utilized information from community registers, parents, teachers and classmates in order to investigate a possible bias in association measures and prevalence estimates. Responders were compared with late responders and non-responders, demonstrating that extra efforts to increase the sample size from 66% to 76% prevented an underestimation of the prevalence of psychopathology. Nonetheless, even with differences between non-responders and responders on several individual characteristics, no significant differences were found pertaining to associations between these characteristics and psychopathology [4]. In the present study, mental health was associated with lost to follow-up in the western subgroup, and we also detected that after reminders, late responders reported more mental health problems than early responders [49]. For that reason, we may have underreported the occurrence of mental health in the follow-up.

In a two-year follow-up of 15-18-year-old psychiatric outpatients, it was possible to reach all 101 patients except four, using a comprehensive tracking system [26]. Axis I and II disorders at the two-year follow-up were significantly associated with follow-up contact difficulties, while baseline psychopathology and sociodemographic variables were not. Thus, relying on baseline characteristics of adolescents may underestimate the extent of psychopathology at follow-up. Based on the above mentioned studies, there might be a higher rate of mental health problems among those lost to follow-up compared to participants in our study. Even though we found that relying on baseline information yielded a higher overall prevalence of externalized symptoms in those lost to follow-up compared to participants and overall no difference regarding mental distress, we cannot rule out of whether there was any underestimation of psychopathology at follow-up in the present study.

According to Hartge [50], poor response rates may be of little concern if the willingness to participate is essentially unrelated to exposure. Even if willingness differs with exposure, bias will still not result unless the tendency is stronger (or weaker) in different levels of outcome (i.e. in individuals with disease vs. no disease). According to Kleinbaum et al. [51], even if a willingness to participate is unrelated to exposure, this willingness may be stronger (or weaker) associated with baseline exposure by level of outcome, meaning that selection bias may occur. In the present study, we have investigated whether there are differences in associations measures (PRs) between baseline exposures (smoking, physical activity) and baseline health outcomes (self-reported health, mental distress, externalized symptoms) among participants and all invitees. The use of baseline outcome variables must be regarded as a proxy evaluation of selection bias in associations between baseline exposures and outcomes at *follow-up*. In our study, a willingness to participate was associated with one of the two exposure variables, namely baseline smoking, but not with baseline physical activity (Table 4). For the total material and in Norwegian/western participants, we detected no selection bias in the association measures (PRs) when utilizing baseline smoking and physical activity as the exposures and selected baseline outcomes (mental distress, externalized symptoms, self-reported health), thereby indicating that the associations between willingness to participate and smoking (and physical activity) are similar by level of outcomes [51]. However, in subgroups of non-western youths (especially in boys) the association between willingness to participate and exposure (particularly with smoking) differed by level of outcome (mental distress, externalized symptoms, self-reported health). So in accordance with Kleinbaum [51], the estimated PRs are biased in these subgroups of participants due to selection, which are indicated with different estimates of PRs between participating and all invited non-western boys and girls (Table 6). However, the number of participants in these groups was low and the confidence intervals were wide.

Unfortunately, it is not possible to conclude that the association between *baseline exposures *(smoking or physical activity) and selected outcomes measured at *follow-up *(self-reported health, mental distress, externalized symptoms) are free from selection bias, but if we lean on analyses of the present baseline outcome data and reports from previous studies [4,6], we may be able to say that there is probably no major selection bias. Regarding subgroups of non-western immigrants, it is more likely that the association measure is biased.

### DNA

In the present study, it is not possible to directly assess whether the task of providing DNA has affected the rate of lost to follow-up among 18/19 year olds. However, in the school-based study of 13th graders in which DNA was provided, the response rate was high at 90%, which is similar to the school-based surveys of 10th graders in which DNA sampling was not included. Because of this, it is unlikely that a particular fear of providing DNA played a role in the response rate for the school-based portion of the study. In a qualitative study in the UK [52] of 23-67-year-old participants from an epidemiological health study which collected DNA, it was reported that most of the panel had a positive attitude to medical research and that genetic research in particular was seen as being especially rich in the potential for medical advancement. Other reasons for participating in this genetic health study were: a desire to do good; the possibility of a health gain in the form of a health check; confidence in the research process and its governance and a perception of low risk [52]. The study revealed that the participants had these positive attitudes although most of them misunderstood the aim of the genetic epidemiological study, which was explained in information leaflets. It is not known which factors were in operation for adolescents, and this should be further explored.

To the best of our knowledge, the present study is the first of its kind to investigate predictors of *failure to provide DNA*. We hypothesize from the present data that there are similar personal reasons behind a willingness to provide DNA and a willingness to agree to linkage of data to registers and health surveys, which should also be further explored. The proposed hypothesis is based on detection of a similarly high response rate in the school-based studies that *did or did not *collect DNA, with a consent rate as high for providing DNA as for linking data to registers and health surveys.

## Conclusions

Studies on the effect of self-selection on association measures in epidemiological studies on adolescents are scarce. For this reason, more studies primarily designed to address this topic are needed. Carefully designed studies on self-selection problems may, however, not be a universal answer or truth for all other studies on adolescents. Consequently, epidemiological studies should be carefully planned to allow a judgement of strength and direction of potential errors due to selection. In the present study, associations between selected exposures and health variables measured by prevalence ratios differed somewhat between the participants and all the invitees for non-western boys and girls, although not for western boys and girls. Further studies that aim at validating instruments and questions in a multicultural setting are recommended, as the difference between ethnic groups could be due to linguistic and cultural differences. In general, however, we conclude that the estimated prevalence ratios in the present study were only marginally influenced by lost to follow-up.

As opposed to most studies on adolescents, we did not find a lower response rate in urban as compared with a rural area, and we conclude with similar ratios among 15/16-year-old adolescents in the school-based studies in urban Oslo and rural Hedmark.

As expected, the response rate was considerably higher in school-based surveys than in postal-based surveys. It is not likely that the invitation to provide DNA has influenced the response rates of 18/19 year olds, especially in the school-based survey. We also conclude that the willingness to provide DNA is slightly lower in a postal-based study as compared with a school-based study and that there were similar proportions of participants who consented to provide DNA and who agreed to the linkage of data to other health surveys and health registers. Non-western ethnicity, male gender and characteristics related to low social class and general and mental health problems measured at baseline were associated with lost to follow-up and failure to provide DNA in the present genetic epidemiological study. The predictors were similar to those of non-genetic epidemiological studies of adolescents.

This study is based on both urban and rural samples, and results regarding lost to follow-up, failure to provide DNA and self-selection on association measures were similar across the various samples. The findings may therefore be generalizable to adolescents living in similar urban and rural areas. However, care should be taken in generalizing findings from the group of non-western ethnicity due to low numbers and corresponding wide confidence intervals of estimates.

## Competing interests

The authors declare that they have no competing interests.

## Authors' contributions

EB was the project manager and responsible for the conception of the follow-up project, conceived the present study, took part in the design of the study and interpretation of the data, and drafted the manuscript. ÅS took part in the planning and design of the follow-up project, coordinated the practical part of the project, took part in the data collection and design of the study, discussed the analysis and interpretation of the data, and reviewed the article critically. KG and LL took part in the planning, design and data collection of the study, discussed the analysis and interpretation of the data, and reviewed the article critically. AJS was project manager of the baseline study in Oslo, participated in the planning and design of the follow-up project, took part in the design of the study, discussed the analysis and interpretation of the data, and reviewed the article critically. RS took part in the collecting of the baseline data, carried out all the statistical analyses of the study, took part in the planning and design of the study as well as the interpretation of the data, and reviewed the article critically. All authors read and approved the final manuscript.

## Pre-publication history

The pre-publication history for this paper can be accessed here:

http://www.biomedcentral.com/1471-2458/10/602/prepub
